# miR-25 promotes hepatocellular carcinoma cell growth, migration and invasion by inhibiting RhoGDI1

**DOI:** 10.18632/oncotarget.4740

**Published:** 2015-10-10

**Authors:** Congren Wang, Xuejin Wang, Zijian Su, Hongjiang Fei, Xiaoyu Liu, Qunxiong Pan

**Affiliations:** ^1^ Department of Surgical Oncology, First Hospital of Quanzhou Affiliated to Fujian Medical University, Quanzhou 362000, China; ^2^ Department of Obstetrics and Gynecology, Second Affiliated Hospital of Fujian Medical University, Quanzhou 362000, China

**Keywords:** miR-25, hepatocellular carcinoma, RhoGDI1, metastasis

## Abstract

MicroRNA (miRNA)-25 is a small non-coding RNA that has been implicated in the tumorigenesis of many cancers, but little is known on the role of miR-25 in HCC metastasis. We hereby found that miR-25 was significantly upregulated in clinical HCC tissues compared with normal liver tissues. We also revealed that miR-25 dramatically stimulates HCC cell growth and activates the epithelial-mesenchymal transition (EMT). MiR-25 is activated by the WNT/β-catenin signaling pathway, and exerts its pro-metastatic function by directly inhibiting the Rho GDP dissociation inhibitor alpha (RhoGDI1). Downregulation of RhoGDI1 enhances expression of Snail, thereby promoting EMT. MiR-25 levels are positively correlated with β-catenin expression, whereas negatively correlated with the level of RhoGDI1 in HCC. Our findings provide new insights into the role of miR-25 in HCC metastasis, and implicate the potential application of miR-25 in HCC therapy.

## INTRODUCTION

In the treatment of hepatocellular carcinoma (HCC), high recurrence and metastasis rate contribute to low survival rate for patients [1], and molecular mechanisms underlying HCC metastasis remains largely unknown. MicroRNAs (miRNA) comprise a family of small, noncoding RNA molecules that play important roles in cancer initiation and progression. MiRNAs undergo aberrant regulation during carcinogenesis, leading to therapeutic resistance and metastasis in many cancers [2]. For example, miR-31, miR-135b, and miR-494 are functionally associated with cancer cell proliferation, invasion, and metastasis [3–5], and inhibition of these miRNAs can suppress cancer progression, indicating that miRNAs may be an effective therapeutic target for cancer treatment.

MiR-25 was reported to be associated with tumor carcinogenesis, including breast cancer [6], cholangiocarcinoma [7] and ovarian cancer [8]. High levels of miR-25 in plasma of patients with gastric cancer were associated with the metastasis [9]. However the role of miR-25 in HCC metastasis and invasion remains unknown. Convincing evidences revealed that miR-25 was strongly correlated with tumor metastasis, such as in esophageal carcinoma [10] and gastric cancer [11]. Therefore, we speculated that miR-25 might stimulate the metastasis of HCC cells.

In this study, we demonstrate that miR-25 is upregulated and correlated with the progression of the tumor in HCC. MiR-25 is activated by WNT/β-catenin signaling to promote HCC cell EMT, migration and invasion, through direct targeting of the Rho GDP dissociation inhibitor alpha (RhoGDI1) both *in vitro* and *in vivo*. Downregulation of RhoGDI1 enhances expression of Snail, transcriptional repressor of E-cadherin, thereby promoting EMT. Our data provide new insights into the role of miR-25 in metastasis as well as its regulatory mechanisms in HCC.

## RESULTS

### Upregulation of miR-25 is correlated with the progression of HCC

**Figure 1:**
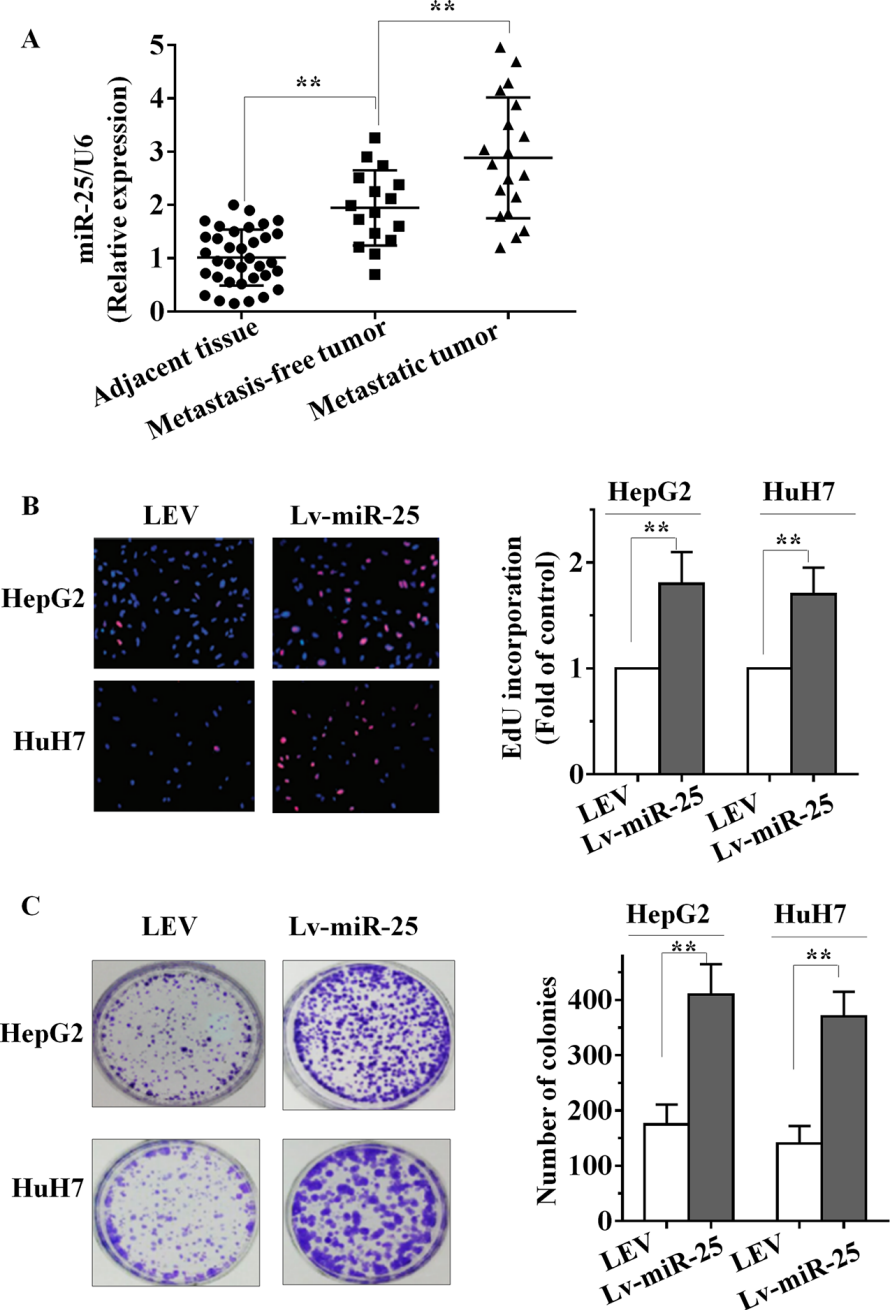
miR-25 is upregulated in HCC tissues and promotes HCC cell growth *in vitro* **A.** Increased levels of miR-25 were positively correlated with the tumor grades and metastasis of HCC by qRT–PCR. miR-25 expression levels were calculated by the miR-25/U6 expression ratio (2^−ΔΔCT^). **B–C.** The effect of Lv-miR-25 or LEV on the growth of HepG2 and HuH7 cells was examined by EdU incorporation assay (B) and colony formation assay (C). Data are presented as mean ± SD from three independent experiments. **P < 0.01 compared with the control group.

To determine the expression level of miR-25 in HCC, quantitative real-time RT-PCR (qRT-PCR) analysis was performed in 35 pairs (19 pairs were collected from lymph node and distant metastases patients, another 16 pairs from metastasis-free patients) of snap-frozen human primary HCC and corresponding adjacent liver specimens. As shown in Figure 1A, we determined that miR-25 expression in primary HCC tissues was significantly higher than that observed in pair-matched adjacent nontumourous tissues (*p* < 0.001). The parameters of 35 HCC patients were summarized in Table 1. Compared with those without lymph node metastasis, miR-25 expression was significantly higher in those HCC patients with lymph node metastasis (*p* = 0.002). In association with HCV or HBV, HCC patients with positive expression of HCV or HBV had a higher level of miR-25 than those with negative expression. In contrast, no statistically significant relations were detected between miR-25 expression and factors such as age, gender, tumor size, portal vein invasion, AFP and HCC cirrhosis (Table 1). These results suggest that upregulation of miR-25 is correlated with the progression of HCC, as well as a correlation between miR-25 and HCC metastasis.

**Table 1:** Correlation of miR-25 expression in HCC with 35 patients' clinical and pathological properties

Clinicopathological features	*n*	Percentage	miRNA-25 relevant expression (2 −ΔCq)	
			Mean ± SD	*p*
Tissue				
Adjacent non-cancerous liver	35	100%	1.01 ± 0.53	<0.001*
HCC	35	100%	2.46 ± 1.06	
Age				
≥50	22	62.9%	2.3 ± 1.12	0.27
<50	13	37.1%	2.71 ± 0.93	
Gender				
Male	20	57.1%	2.37 ± 1.16	0.57
Female	15	42.9%	2.57 ± 0.93	
Size				
<5 cm	21	60.0%	2.32 ± 1.15	0.35
≥5 cm	14	40.0%	2.66 ± 0.90	
Metastasis				
Without metastasis	16	45.7%	1.94 ± 0.71	0.002*
With metastasis	19	54.3%	2.88 ± 1.13	
Portal vein tumor embolus				
−	18	51.4%	2.51 ± 1.15	0.78
+	17	48.6%	2.40 ± 0.99	
HCV				
−	22	62.9%	2.28 ± 1.14	0.19
+	13	37.1%	2.75 ± 0.87	
HBV				
−	14	40.0%	2.02 ± 0.72	0.033
+	21	60.0%	2.7 ± 1.17	
AFP				
−	19	54.3%	2.43 ± 1.16	0.88
+	16	45.7%	2.49 ± 0.96	
Cirrhosis				
−	17	48.6%	2.36 ± 1.00	0.61
+	18	51.4%	2.54 ± 1.13	

Abbreviations: AFP, alpha-fetoprotein.

* Means *p* < 0.05.

### Overexpression of miR-25 promotes growth of HCC cells *in vitro*

To identify its role in HCC, miR-25 overexpression lentiviral vector (Lv-miR-25) and a control lentiviral empty vector [5] were stably transfected into human HCC HepG2 and HuH7 cells. More than 200-fold increase in miR-25 expression was observed in Lv-miR-25-HepG2 cells and Lv-miR-25-HuH7 cells, compared with the LEV group by qRT-PCR (Supplementary Figure S1A and S1B). As shown in Figure 1B, EdU incorporation assays showed that the proliferation of Lv-miR-25-HepG2 and Lv-miR-25-HuH7 cells was significantly promoted by Lv-miR-25 compared to LEV control. In addition, ectopic miR-25 expression increased growth of HCC cells in colony formation assays (Figure 1C, *p* < 0.01). These observations indicate that miR-25 promotes growth of HCC cells *in vitro*.

### MiR-25 expression enhances migratory, invasive ability and activates EMT in HCC cells

**Figure 2:**
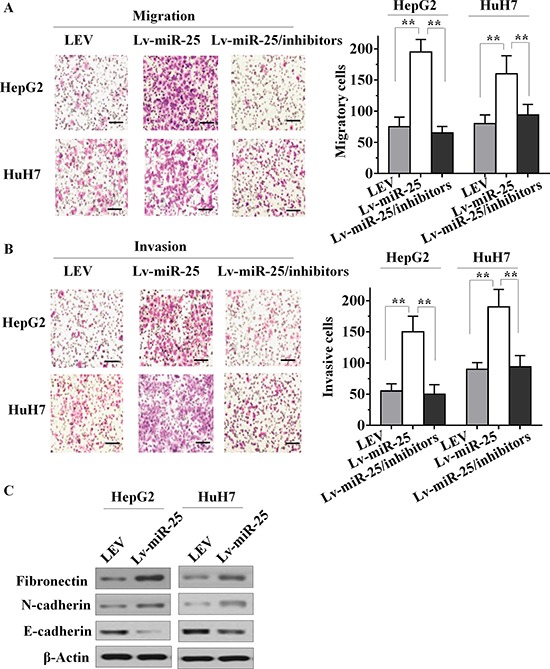
Effect of miR-25 on HCC cell motility, invasion and EMT *in vitro* **A.** Stably upregulating miR-25 increased the migration ability of HepG2 and HuH7 cells *in vitro*, and knockdown of miR-25 in Lv-miR-25 cells reduced the migration ability. **B.** Stably upregulating miR-25 induced *in vitro* invasiveness of HepG2 and HuH7 cells, and specific inhibition of miR-25 in Lv-miR-25 cells reduced the invasion ability. **C.** Immunoblotting of fibronectin, N-cadherin, and E-cadherin in HepG2 and HuH7 cells. Data are presented as mean ± SD from three independent experiments. **p < 0.01 compared with the control group. Scale bar, 100 μm.

Since miR-25 expression is closely associated with aggressive HCC features and metastatic properties, we postulated that miR-25 could have an important role in HCC cells metastasis. To test this hypothesis, we determined changes in cell migration after 6 h of incubation using a transwell chamber. Compared with the LEV cells, Lv-miR-25-HepG2 and Lv-miR-25-HuH7 HCC cells both showed significantly increased migratory ability (both *p* < 0.01, Figure 2A). In addition, Lv-miR-25 and LEV cells were cultured in a Boyden chamber. After 8 h of incubation, Lv-miR-25-HepG2 and Lv-miR-25-HuH7 cells invaded through the matrigel, with a significant increase observed compared with that of control LEV cells (both *p* < 0.01; Figure 2B). Moreover, a significant decrease of cell migration and invasion was found in Lv-miR-25/inhibitor group compared with the Lv-miR-25 group, although there was no significant difference between miR-25 inhibitor group and NC group. We also tested the role of miR-25 in HCC metastasis using wound healing assays, and the results further confirmed that miR-25 overexpression enhances migratory ability (Supplementary Figure S2A and S2B). Furthermore, miR-25 significantly reduced the E-cadherin, but increased the fibronectin levels (Figure 2C), a hallmark of the mesenchymal phenotype. Taken together, these *in vitro* results suggest that miR-25 promotes cancer cell migration, invasion and induces EMT.

### MiR-25 directly targets the RhoGDI1 3′-UTR

**Figure 3:**
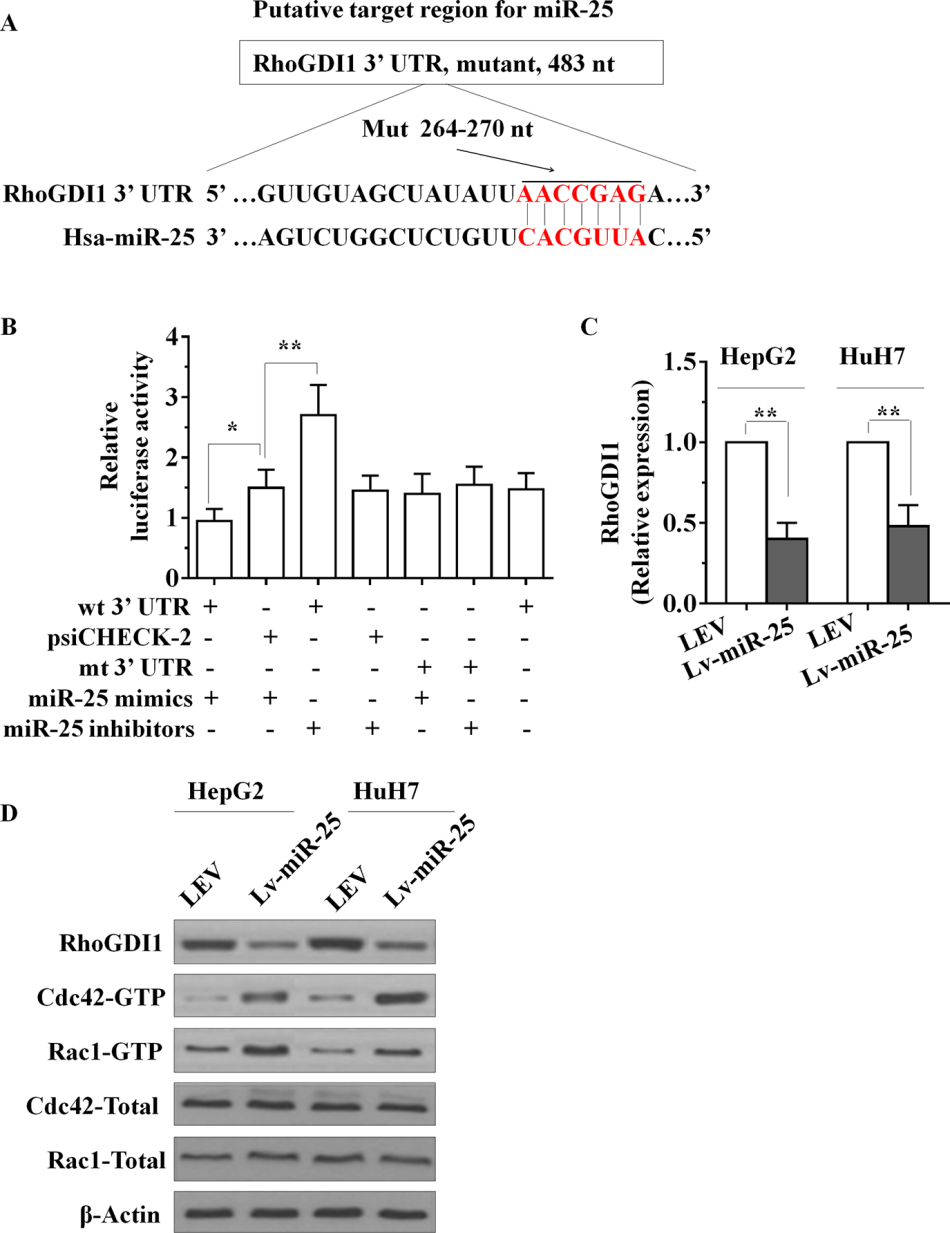
miR-25 directly targets the RhoGDI1 via its 3′-UTR **A.** Sequence alignment between miR-25 and the 3′-UTR of human RhoGDI1 mRNA, nt, nucleotides. **B.** The effect of miR-25 on the activity of firefly luciferase reporter containing either wild type (WT) or mutant type (Mut) 3′-UTR was tested using luciferase reporter gene assays. **C.** The effect of miR-25 on the endogenous expression levels of RhoGDI1 was examined in HepG2 and HuH7 cells by qRT–PCR. **D.** Protein levels of RhoGDI1, the activities of Rac1 and Cdc42 in HepG2 and HuH7 cells were determined after transfection with Lv-miR-25 or LEV by Western blot analysis. Data are presented as mean ± SD from three independent experiments. *p < 0.05, **p < 0.01 compared with the control LEV group.

To investigate the mechanism by which miR-25 promotes cell proliferation, migration and invasion in HCC cells, we applied two algorithms that predict targets of a miRNA-PicTar [12] and TargetScan [13]. On the basis of the representation of miR-25 sites in their 3′ untranslated regions (UTR), > 100 mRNAs were predicted to be regulated by miR-25. Genes with more than two-fold changes were considered of interest. Among these candidates, six genes (RhoGDI1, ALCAM, FOXJ2, FOXO3, NDRG2 and SOX11) were involved in the suppression of cancer metastasis. Interestingly, we found that RhoGDI1 is the most downregulated among all miR-25 target genes. The putative tumor suppressor RhoGDI1 is an essential component involved in the cell proliferation, migration and invasion in several types of tumors [14, 15]. Little is known about the function of RhoGDI1 in HCC. To confirm whether miR-25 suppressed the expression of RhoGDI1, a dual-luciferase reporter system was employed. We subcloned 3′-UTR region of RhoGDI1 mRNA including the predicted miR-25 recognition site (wild type) or the mutated sequence (mutant type) into luciferase reporter plasmids. Our results showed that the reporter plasmid with 3′-UTR of RhoGDI1 resulted in a significant decrease in luciferase activity after transfection with miR-25 mimic. A significant increase in luciferase activity was observed after transfection with miR-25 inhibitors, whereas the plasmid without RhoGDI1 3′-UTR had no change in luciferase activity (Figure 3A and 3B). mRNA and protein levels of RhoGDI1 in Lv-miR-25-treated cells were detected using qRT-PCR and Western blot. Overexpression of miR-25 significantly decreased both RhoGDI1 mRNA and protein levels compared with LEV cells (Figure 3C and 3D). Taken together, these results suggest that miR-25 downregulates RhoGDI1 expression by directly targeting its 3′-UTR.

### Exogenous expression of miR-25 enhances the activation of Rac and Cdc42

RhoGDI1 is the negative regulator of Rho GTPases such as Rac1 and Cdc42, therefore we next determined whether miR-25 expression changes the activities of Rac and Cdc42. As the PAK-21 protein binds to only activated (GTP-bound) forms of active GTPases, we used a PAK-21 pulldown approach to assay GTPase activity upon transfection of miR-25 mimics. Amounts of GTP-bound Rac1 and Cdc42 were determined by SDS-PAGE followed by immunoblotting by using published methodologies [16]. As seen in Figure 3D, transfection of miR-25 mimics markedly increased the active Rac1 and Cdc42, whereas the total protein levels of Rac1 and Cdc42 were not altered.

### Ectopic expression of RhoGDI1 mitigates miR-25 promotion of HCC cell proliferation, migration and invasion

**Figure 4:**
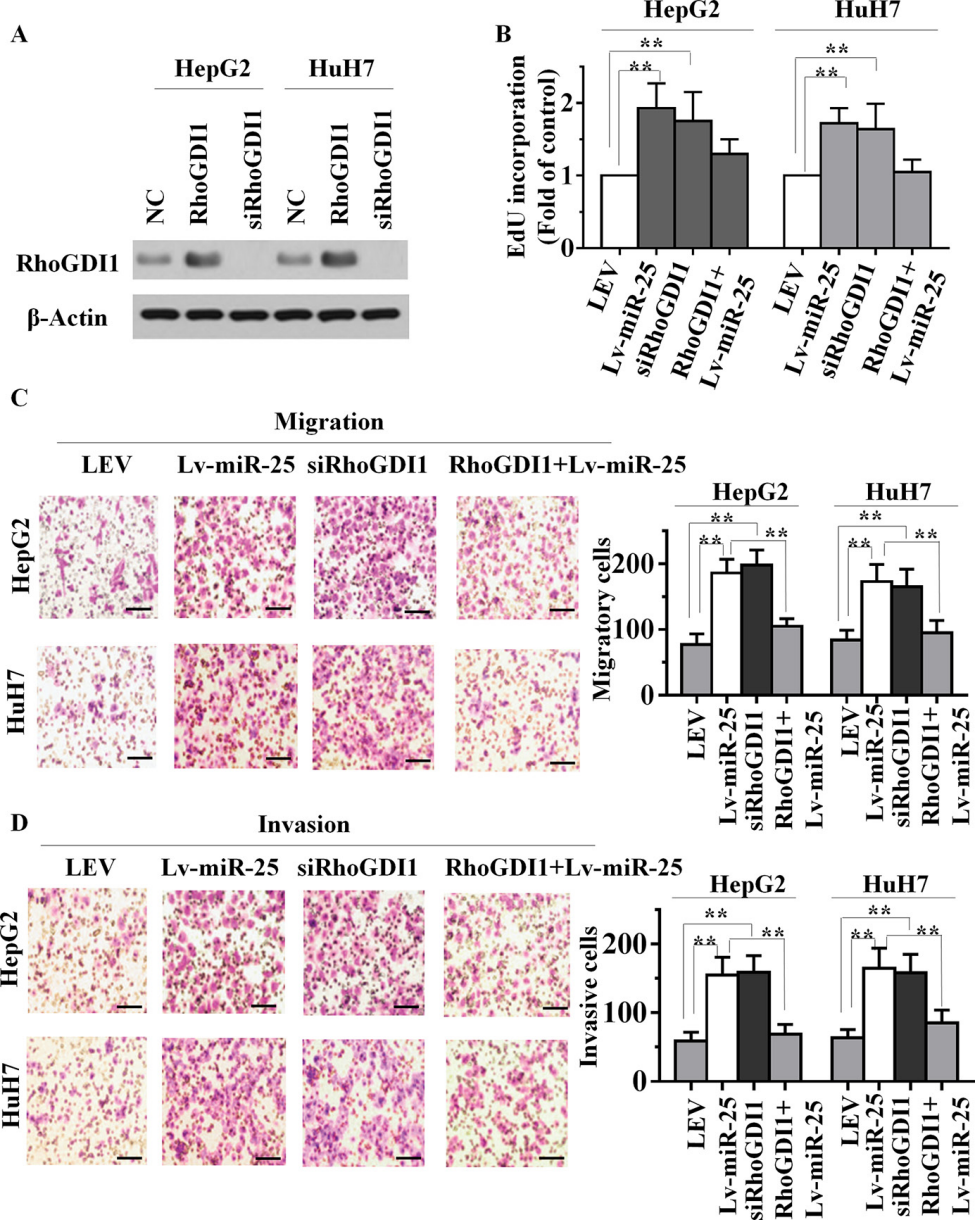
miR-25 overexpression and RhoGDI1 inhibition produce similar changes, which are restored by RhoGDI1 ectopic expression *in vitro* **A.** The expression level of RhoGDI1 was examined by Western blot analyses in HepG2 and HuH7 cells treated with ectopic RhoGDI1 or siRNAs targeting RhoGDI1. EdU incorporation assays **B.** transwell migration assays **C.** and Boyden invasion assays **D.** of HepG2 or HuH7 cells were performed after transfection with NC, Lv-miR-25 and siRNA against RhoGDI1 as indicated. Data are presented as mean ± SD from three independent experiments. **p < 0.01 compared with the control group. Scale bar, 100 μm.

To explore whether miR-25 targeting of RhoGDI1 was responsible for the promotion of the proliferation, migration and invasion of HCC cells, we utilized an expression construct that encodes the entire RhoGDI1 coding sequence but lacks its 3′-UTR. We inserted this sequence into a plasmid (plasmid-RhoGDI1), and simultaneously suppressed RhoGDI1 with specific small-interfering RNA (siRNA) - siRhoGDI1. The expression of RhoGDI1 protein in plasmid-RhoGDI1-treated cells was significantly upregulated compared with cells transfected by empty vector. Western blot analysis also demonstrated that siRhoGDI1 was able to effectively knockdown the expression of RhoGDI1 in HepG2 and HuH7 cells (Figure 4A). Further functional studies demonstrated that knockdown of RhoGDI1 produced similar changes in the proliferative, migratory and invasive capacity assay compared with those of Lv-miR-25 groups (Figure 4B, and 4D). We further confirmed these results using wound healing assays (Supplementary Figure S2A and S2B). Ectopic expression of RhoGDI1 with plasmid-RhoGDI1 partially mitigated miR-25-mediated induction of growth, migration and invasion. This suggested that the effects of miR-25 overexpression in HCC cells depend specifically on RhoGDI1 suppression.

### MiR-25 inhibits RhoGDI1 to promote metastasis

**Figure 5:**
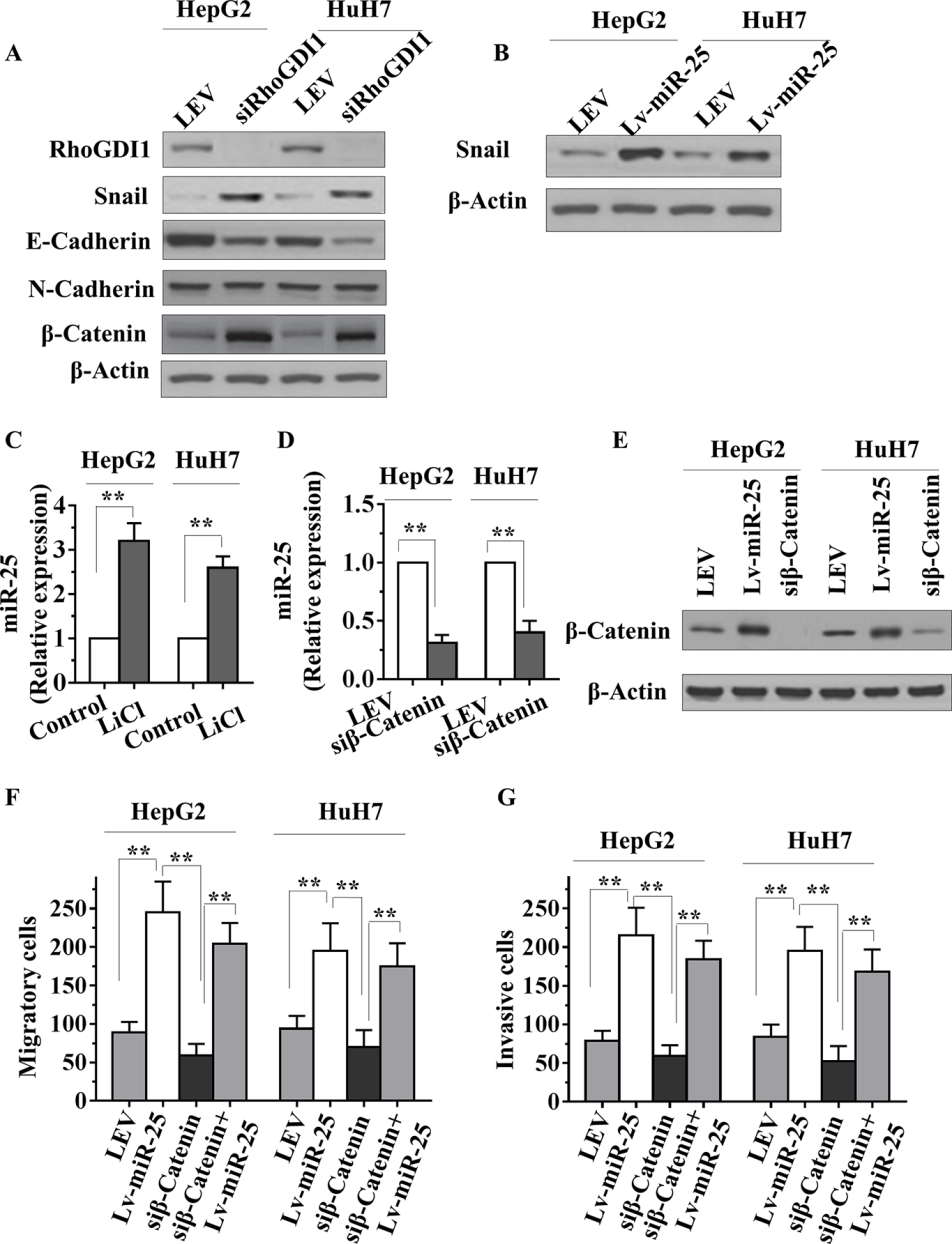
miR-25 inhibits RhoGDI1 and is activated by WNT/β-catenin signaling in HCC cells **A.** HepG2 or HuH7 cells were transfected with RhoGDI1 siRNAs, then RhoGDI1, Snail, E-cadherin, N-cadherin and β-catenin protein levels were detected by Western blot analysis. **B.** HepG2 or HuH7 cells were transfected with negative control (NC), miR-25 mimics, or miR-25 inhibitor, and Snail was then detected by Western blot analysis. **C.** qRT-PCR analysis of miR-25 in HepG2 and HuH7 cells treated with or without LiCl (20 mmol/L) for 36 hours. **D–E.** qRT-PCR analysis of miR-25 and Western blot analysis of β-catenin protein levels in HepG2 and HuH7 cells transfected with NC or siRNAs against β-catenin. qRT-PCR data were normalized using U6 RNA. **F–G.** Transwell migration (F) and Boyden invasion (G) assays were performed in HepG2 and HuH7 cells transfected with NC, Lv-miR-25, and/or siRNAs against β-catenin. Data are presented as mean ± SD from three independent experiments. **p < 0.01 compared with the control group.

We next asked whether RhoGDI1 connects miR-25 and EMT. Interestingly, knockdown of RhoGDI1 significantly reduced E-cadherin, whereas increased N-cadherin levels (Figure 5A). Loss of E-cadherin is the hallmark of EMT, and several transcription factors have been implicated in the transcriptional repression of E-cadherin. Snail is the most important transcriptional repressor of E-cadherin [17], and was found to promote tumor progression in HCC [18, 19]. We found that Snail was upregulated by miR-25 (Figure 5B). Interestingly, knockdown of RhoGDI1 produced a similar change in Snail expression to that of miR-25 (Figure 5A and 5B). Taken together, these data indicated that the ability of miR-25 to promote metastasis is attributable, in a significant part, to its capacity to inhibit RhoGDI1.

### MiR-25 is activated by WNT/β-catenin signaling in HCC cells

MiR-25 expression in oesophageal squamous cell carcinoma cells can be driven by β-catenin [20]. To further confirm these results in HCC cells, we stabilized β-catenin protein by treating HepG2 and HuH7 cells with lithium chloride (LiCl), an inhibitor of GSK3β, which is responsible for β-catenin degradation. We found that miR-25 expression was markedly activated by LiCl treatment in both cell lines (Figure 5C). Interestingly, a significant reduction of miR-25 expression in cells transfected with β-catenin siRNA was verified by qRT-PCR (Figure 5D and 5E). We next asked whether RhoGDI1 connects β-catenin, and found that β-catenin was increased by knockdown of RhoGDI1 (Figure 5A). Knockdown of β-catenin was reported to reduce the invasiveness of cancer cell [21]. The migration and invasion assay results showed that knockdown of β-catenin decreased the migratory and invasiveness of HepG2 and HuH7 cells. Furthermore, reexpression of miR-25 restored the decreased migratory and invasiveness of cells (Figure 5F and 5G). Taken together, these results suggest that miR-25 expression can be driven by WNT/β-catenin signaling, and may play a role in WNT/β-catenin-mediated metastasis.

### The overexpression of miR-25 promotes HCC growth and metastasis *in vivo*

**Figure 6:**
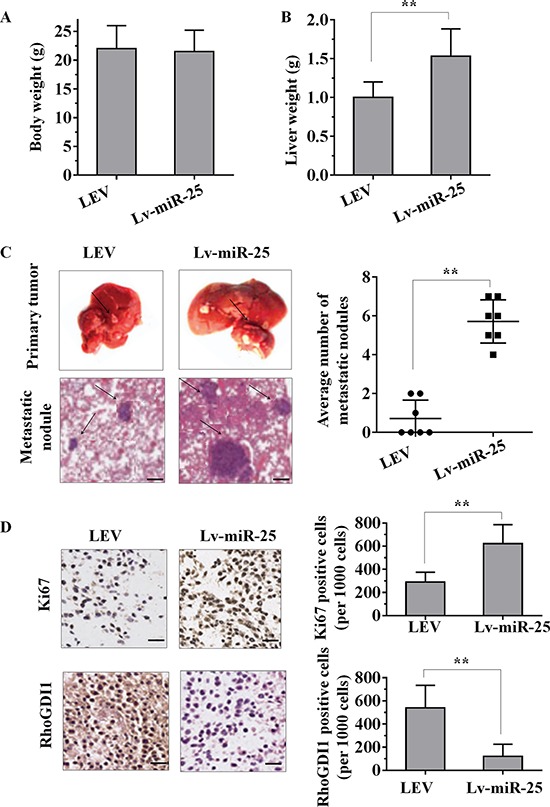
The overexpression of miR-25 promotes tumour growth and metastasis *in vivo* **A–B.** Female nude mouse was orthotopically inoculated in the left hepatic lobe with Lv-miR-25-HepG2 or LEV-HepG2 cells. Six weeks later, the body weights (A) and liver weights (B) of the mice were measured. **C.** Representative images of tumour nodules in primary sites, and metastatic nodules were present at the sixth week after the orthotopic liver inoculation of nude mice. The black arrows indicate the location of primary tumour nodules and metastatic nodules. The number of metastatic nodules in each mouse was counted. **D.** Representative immunohistochemical staining sections of primary liver tumours formed by Lv-miR-25-HepG2 or LEV-HepG2. For each generated tumour, five fields were randomly selected. The number of Ki67-positive and RhoGDI1-positive cells per 1000 cells was counted by three independent experienced pathologists. Data are presented as mean ± SD. **p < 0.01 compared with the control group. Scale bar, 100 μm.

Given that miR-25 promoted the cell proliferation, migration and invasion of HCC cells *in vitro*, we further investigated whether miR-25 could stimulate tumor growth and metastasis *in vivo*. Lv-miR-25-HepG2 or LEV-HepG2 cells were orthotopically inoculated into the left hepatic lobe livers of nude mice for intrahepatic and distant metastasis. After 6 weeks of inoculation, body weights of the mice did not differ (Figure 6A). However, liver weight of the Lv-miR-25 group were significantly higher than that of the LEV group (Figure 6B). All of the mice (*n* = 7) inoculated with Lv-miR-25-HepG2 cells developed remarkable metastatic nodules. In contrast, only three of the mice (*n* = 7) inoculated with LEV-HepG2 cells had metastatic nodules (Figure 6C). Moreover, Ki-67 staining showed that tumors of Lv-miR-25-HepG2 cells had more proliferative cells than LEV-HepG2 tumor cells (Figure 6D). We also consistently observed the downregulation of RhoGDI1 in tumors generated from Lv-miR-25-HepG2 cells compared with those generated from LEV-HepG2 cells, as demonstrated by immunohistochemical analysis (Figure 6D). Taken together, these observations suggest that miR-25 may promote tumor metastasis by downregulating RhoGDI1 *in vivo*.

## DISCUSSION

We hereby demonstrated that miR-25, which is upregulated by WNT/β-catenin signaling, promotes EMT and hepatocellular carcinoma (HCC) metastasis through direct targeting of RhoGDI1 both *in vitro* and *in vivo*. We found that downregulation of RhoGDI1, a direct and functional target of miR-25, markedly increases Snail expression thereby promoting EMT. Taken together, our findings indicate that upregulation of miR-25 has an important role in the process of HCC metastasis, and miR-25 may be a novel potential therapeutic target for HCC treatment.

MiR-25 associates with tumor carcinogenesis, and its overexpression blocks isoliquiritigenin-induced autophagy and chemosensitization in breast cancer cells [6]. MiR-25 also promotes apoptosis resistance in cholangiocarcinoma by targeting TRAL death receptor-4 [7], as well as ovarian cancer proliferation and motility by targeting LATS2 [8]. We confirmed that miR-25 was upregulated in HCC, consistent with previous report [22]. We also found that miR-25 expression levels were higher in patients with distant metastases than metastasis-free patients, implying a role of miR-25 overexpression in HCC metastasis. Apart from overexpression, miR-25 is nevertheless downregulated in the other types of human tumors. Li showed that miR-25 was reduced in colon cancer, and restoration of miR-25 inhibited cell proliferation and migration [23]. Esposito demonstrated that miR-25 was decreased in anaplastic thyroid carcinomas, and ectopic expression of miR-25 suppressed the proliferation and colony formation of anaplastic thyroid carcinoma cells by inducing G2/M-phase cell cycle arrest [24]. Therefore miR-25 expression may be differentially regulated in the development of different tumors. Recently, possible function of miR-25 in tumor progress was reported to be associated with tumor metastasis and invasion. Aberrant expression of miR-25 correlated with tumor metastasis and invasion, for instance, Xu showed that miR-25 promoted cell migration and invasion in esophageal squamous cell carcinoma [10]. Kim demonstrated that the overexpression of miR-25 in gastric cancer tissues was associated with lymph node metastasis [11]. Recently Li provided evidence that miR-25 promotes gastric cancer migration, invasion and proliferation by directly targeting transducer of ERBB2, 1 [25]. Consistent with these findings, our study showed that miR-25 promoted the cell proliferation, migration and invasion of HCC cells *in vitro* and *in vivo*. Our study indicated miR-25 may be a potential prognostic biomarker for its application in clinical practice. These findings open a novel avenue to investigate the molecular mechanism of HCC progression and to develop potential therapeutics against HCC. However, no studies have reported a definitive mechanism for the upregulation of miR-25 in HCCs, which requires further exploration. Here we identified miR-25 as a prometastatic miRNA and a negative regulator of the key metastasis suppressor RhoGDI1 in HCC.

Dysregulation of the Wnt/β-catenin pathway has been observed in various cancers. It was recently shown that altered β-catenin expression is associated with poor prognosis in HCC patients [26, 27]. We demonstrated that Wnt/β-catenin signaling activates miR-25 to promote EMT and enhance invasiveness and motility of HCC cells. Level of RhoGDI1 is also negatively correlated with β-catenin expression. Therefore our study revealed a novel mechanism by which Wnt/β-catenin signaling contributes to cancer metastasis. Previous studies have reported that Rho GTPases contribute to multiple cellular processes that could stimulate cancer progression, including cytoskeletal dynamics, cell adhesion, and migration [28]. Recent studies indicate that RhoGDI1 functions as a candidate metastasis suppressor, which is frequently downregulated in HCC, breast cancer and lung cancer [15, 29, 30]. Our findings showed that miR-25 directly suppresses RhoGDI1 expression and activates Rac1 and Cdc42, which in turn increases cell motility, invasiveness and metastasis. We also found that downregulation of RhoGDI1 induced Snail expression and promoted EMT, which further establishes RhoGDI1 as a critical metastasis suppressor.

In conclusion, our study demonstrated that overexpression of miR-25 is able to promote HCC cell proliferation, invasion and metastasis. MiR-25 is activated by the WNT/β-catenin signaling pathway and exerts its pro-metastatic function by directly inhibiting RhoGDI1. Downregulation of RhoGDI1 enhances the induction of Snail and promotes EMT. MiR-25/RhoGDI1 axis provides new insight into the pathogenesis of HCC, particularly with respect to invasion and metastasis, and represents a potential therapeutic target for the treatment of HCC.

## MATERIALS AND METHODS

### Cell culture

HCC cell lines, HepG2 and HuH7 were purchased from American Type Culture Collection (Rockville, MD), and cultured as recommended as monolayers in RPMI-1640 supplemented with 10% heat-inactivated fetal bovine serum (Hyclone, Logan, UT), penicillin (100 U/mL)/streptomycin (100 μg/mL)/amphotericin B (0.25 μg/mL) from Invitrogen (Carlsbad, CA) in a humidified incubator at 37°C in a 5% CO_2_ atmosphere.

### Tissue samples

A total of 35 pairs of human primary HCC tissues and their corresponding nontumourous liver specimens were collected from The First Hospital of Quanzhou Affiliated to Fujian Medical University, China. Among these specimens, 19 pairs of HCC and corresponding adjacent nontumourous liver tissues were collected from patients with lymph node and distant metastases. Another 16 pairs of fresh HCC and adjacent liver tissues were collected from metastasis-free patients. These HCC cases were from 20 males and 15 females and none of the patients had received radiotherapy or chemotherapy before surgery. The clinicopathological characteristics (including age, tumor size and other information) were presented in Table 1. For the use of these clinical materials for research purposes, prior consent from patients and approval from the Ethics Committee of The First Hospital of Quanzhou Affiliated to Fujian Medical University were obtained. The subject gave informed consent. Patient anonymity has been preserved. All specimens had confirmed pathological diagnosis and were classified according to the WHO (World Health Organization) criteria.

### RNA isolation, reverse transcription and qRT-PCR

Total RNA was extracted from the HCC tissues and adjacent nontumourous liver tissues by Trizol Reagent (Invitrogen, Carlsbad, CA, USA). For miR-25 detection, reverse-transcribed complementary DNA was synthesized with the PrimeScript RT reagent Kit (TaKaRa, Dalian, China), and quantitative real time-PCR (qRT-PCR) was performed with SYBR Premix ExTaq (TaKaRa, Dalian, China) with the Stratagene Mx3000P real-time PCR system (Agilent Technologies, Inc., Santa Clara, CA, USA). Expression levels were normalized against the endogenous snRNA U6 control. The relative expression ratio of miR-25 in each paired tumour and nontumourous tissue was calculated by the 2^−ΔΔCT^ method. For mRNA analyses, cDNA was synthesized using Moloney murine leukaemia virus reverse transcriptase (Promega, Madison, WI, USA). qRT-PCR was performed with SYBR Premix ExTaq with the Stratagene Mx3000P real-time PCR system. β-actin was used as internal controls for mRNA quantification. The relative expression ratio of mRNA was calculated by the 2^−ΔΔCT^ method. PCR reactions for each gene were repeated three times. Independent experiments were done in triplicate. All the sequences of the PCR primers and other oligonucleotides used in this study are shown in Supplementary Table 1.

### Establishment of HCC cell lines with stable expression of miR-25

Lentiviral vectors which overexpress miR-25 were purchased from GeneChem (Shanghai, China). A lentiviral vector expressing scrambled RNA was used as the control and the sequence was 5′-TTCTCCGAACGTGTCACGT-3′. HepG2 or HuH7 cells were infected with lentiviral vector, and polyclonal cells with green fluorescent protein signals were selected for further experiments using fluorescence-activated cell sorting flow cytometry. Total RNA from these cell clones was isolated, and levels of miR-25 were quantified using qRT-PCR.

### EdU proliferation assay

The proliferation of HepG2 and HuH7 cells was examined using the Cell-Light EdU Apollo488 *In Vitro* Imaging Kit (RiboBio) according to the manufacturer's protocol. Briefly, cells were incubated with 10 μM EdU for 2 h before fixation with 4% paraformaldehyde, permeabilization by 0.3% Triton X-100 and EdU staining. Cell nuclei were stained with 5 μg/mL DAPI (4′, 6-diamidino-2-phenylindole) for 10 min. The number of Edu-positive cells was counted under a microscope in five random fields (× 100). All assays were independently performed in triplicate.

### Colony formation assay

HepG2 or HuH7 cells were plated in 6-well culture plates at 200 cells/well. Each cell group had two wells. After incubation for 14 days at 37°C, cells were washed twice with phosphate buffered saline and stained with hematoxylin solution. The number of colonies containing > 50 cells was counted under a microscope. The colony formation efficiency was calculated as (number of colonies/number of cells inoculated) × 100%. All assays were independently performed in triplicate.

### Cell migration and invasion assays

*In vitro* cell migration and invasion assays were examined according to previous study [31]. For the cell migration assay, 1 × 10^4^ cells in 100 μL medium without fetal bovine serum were seeded on a fibronectin-coated polycarbonate membrane insert in a transwell apparatus (Costar, Corning, NY, USA). In the lower chamber, 500 μl medium with 10% fetal bovine serum was added as chemoattractant. After the cells were incubated for 6 h at 37°C in a 5% CO_2_ atmosphere, the insert was washed with phosphate buffered saline, and cells on the top surface of the insert were removed with a cotton swab. Cells adhering to the lower surface were fixed with methanol, stained with crystal violet solution and counted under a microscope in five predetermined fields (× 100). All assays were independently repeated at least thrice. The procedure for the cell invasion assay was similar to the cell migration assay, except that the transwell membranes were precoated with 24 μg/μL matrigel (R&D Systems, Inc., Minneapolis, MN, USA) and the cells were incubated for 8 h at 37°C in a 5% CO_2_ atmosphere. Cells adhering to the lower surface were counted the same way as the cell migration assay.

### Wound-healing assay

HepG2 or HuH7 cells (1 × 10^6^ cells per well) were seeded on 6-well plates. Twenty-four hours later, the cells were transfected with siRNA for RhoGDI1 or miR-25 inhibitors. Twenty-four hours after transfection, HepG2 or HuH7 cells were wounded in serum-free medium, 1% bovine serum albumin (BSA) with a sterile 200 μl pipette tip to remove cells. The progress of migration was photographed (after identification of each wounded zone) in six regions, immediately and during 2 days after wounding (0 h–24 h–48 h), using an inverted microscope (Nikon TMS-F, 301655) equipped with a digital camera (Nikon Digital shot DS-L1). Cell migration was expressed as the migration rate: (original scratch width - new scratch width)/original scratch width × 100%.

### Transient transfection with siRNAs or miRNA inhibitors

siRNA for RhoGDI1 and miR-25 inhibitors were designed and synthesized by Guangzhou RiboBio (Guangzhou, China). Three siRNAs targeting RhoGDI1 were designed and synthesized, the most effective siRNA (siRhoGDI1) identified by qRT-PCR was used for further experiments. The sequence of the negative control (NC) was also designed by RiboBio. Twelve hours prior to transfection, cells were plated onto a 6-well or a 96-well plate (Nest Biotech, Shanghai, China) at 30–50% confluence. TurboFect siRNA Transfection Reagent (Fermentas, Vilnius, Lithuania) was then used to transfect siRNA into cells according to the manufacturer's protocol. Cells were collected after 48–72 h for further experiments.

### Western blot analysis

Whole-cell lysates were separated in 12% SDS-PAGE gels and blotted on nitrocellulose membranes, and probed with antibodies against β-Actin (Santa Cruz Biotechnology, Inc.), RhoGDI1, Fibronectin, Snail (Proteintech), N-cadherin (Epitomics), E-cadherin, and β-catenin (Cell Signaling Technology). After incubation with primary antibodies, the membranes were washed with TBS/0.05% Tween-20 and incubated with horseradish peroxidase-conjugated secondary antibodies at room temperature for 1 hour. Signals were detected using enhanced chemiluminescence reagents (Pierce, Rockford, IL, USA).

### Rac1/cdc42 activation assay

Cells were harvested in magnesium-containing lysis buffer 72 h after transfection, and the lysates were sonicated for 5 seconds and centrifuged for 30 minutes at 18, 000 × *g* and 4°C following the manufacturer's specifications for the Rac/Cdc42 Assay Reagent Kit (Upstate Biotechnology, Inc.). A total of 10 μg Rac/Cdc42 assay reagent was added to 600 μL protein lysate and gently rocked at 4°C for 30 minutes. PAK-21-agarose conjugates were collected by centrifugation for 5 seconds at 14, 000 × *g* at room temperature and washed three times with 500 μL magnesium-containing lysis buffer, and bound protein was eluted in 25 μL SDS-PAGE sample buffer. Western blotting of these samples and of 10 μL of the original lysate as a loading control was performed using standard protocols.

### 3′-UTR luciferase reporter assays

For reporter assays, a fragment of RhoGDI1 3′-UTR amplified by PCR primers was cloned into psiCHECK-2 vectors (named wt). Site-directed mutagenesis of the miR-25 binding site in RhoGDI1 3′-UTR was performed using GeneTailor Site-Directed Mutagenesis System (Invitrogen, Guangzhou, China; named mt). wt or mt vector and the control vector psiCHECK-2 vector were cotransfected into HepG2 or HuH7 cells with miR-25 mimics or inhibitors in 48-well plates, and then harvested for luciferase assay 48 h after transfection. Luciferase assays were performed by using the Dual-Luciferase Reporter Assay System (Promega Corporation, Madison, WI, USA) according to the manufacturer's protocol. Firefly luciferase was used for normalization.

### *In vivo* metastasis assays

All procedures and experiments involving animals in this study were performed in accordance with the National Institutes of Health Guide for Care and Use of Laboratory Animals. The protocol was approved by the Committee on the Ethics of Animal Experiments of The First Hospital of Quanzhou Affiliated to Fujian Medical University. All surgery was performed under sodium pentobarbital anesthesia, and all efforts were made to minimize suffering. Female BALB/c-nu/nu mice of 4–5 weeks of age were purchased from Shanghai SLAC Laboratory Animal Co., Ltd. (Shanghai, China) and were housed in the Animal Resource Facility. For *in vivo* metastasis assays, 2 × 10^6^ HepG2 cells transfected with Lv-miR-25 or the control LEV were suspended in 40 μL serum-free Dulbecco's modified Eagle's medium/Matrigel (1:1) for each mouse. Through an 8-mm transverse incision in the upper abdomen under anaesthesia, each nude mouse (seven in each group) was orthotopically inoculated in the left hepatic lobe with a microsyringe. After 6 weeks, the mice were killed, and their livers and lungs were dissected, fixed with phosphate-buffered neutral formalin and prepared for standard histological examination. All studies were performed under the Laboratory Animal Care guidelines for the humane treatment of animals and adhered to national and international standards.

### Immunohistochemistry

Paraffin sections (4 μm thickness) from samples were deparaffinized in 100% xylene and re-hydrated in descending ethanol series and water according to standard protocols. Heat-induced antigen retrieval was performed in 10 mM citrate buffer for 2 min at 100°C. Endogenous peroxidase activity and non-specific antigens were blocked with peroxidase blocking reagent containing 3% hydrogen peroxide and serum, followed by incubation with Ki67 and RhoGDI1 antibody (Cell Signaling Technology, Danvers, MA, USA) overnight at 4°C. After washing, the sections were incubated with biotin-labeled rabbit anti-goat antibody for 15 min at room temperature, and subsequently were incubated with streptavidin-conjugated horseradish peroxidase (Maixin, Fuzhou, China). The peroxidase reaction was developed using 3, 3-diaminobenzidine (DAB) chromogen solution in DAB buffer substrate. Sections were visualized with DAB and counterstained with hematoxylin, mounted in neutral gum and analyzed using a bright field microscope. IHC scores were performed using a semiquantitative grading system as previous study [32]. Sections with no labeling or with < 5% labeled cells were scored as 0. Sections were scored as 1 with labeling of 5–30% of cells, as 2 with 31–70% of cells and as 3 with ≥ 71% of cell. Each sample was examined separately and scored by two blinded pathologists.

### Statistical analysis

Data are presented as mean ± SD unless otherwise indicated. The statistical significance of the difference between the values of control and treatment groups was determined by either Student *t* test or simple one-way ANOVA followed by Tukey's*post hoc* test for multiple comparisons using Prism version 5 (GraphPad Software, Inc.). Values of *p* < 0.05 were considered statistically significant.

## SUPPLEMENTARY FIGURES AND TABLE


