# Supplementary material for: miR-25 promotes hepatocellular carcinoma cell growth, migration and invasion by inhibiting RhoGDI1
Source: Oncotarget. 2015 Oct 10;6(34):36231–44. doi: 10.18632/oncotarget.4740 (PMC4742173; doi:10.18632/oncotarget.4740)
Supplement: Supplementary file 1 [file oncotarget-06-36231-s001.pdf]

SUPPLEMENTARY FIGURES AND TABLE

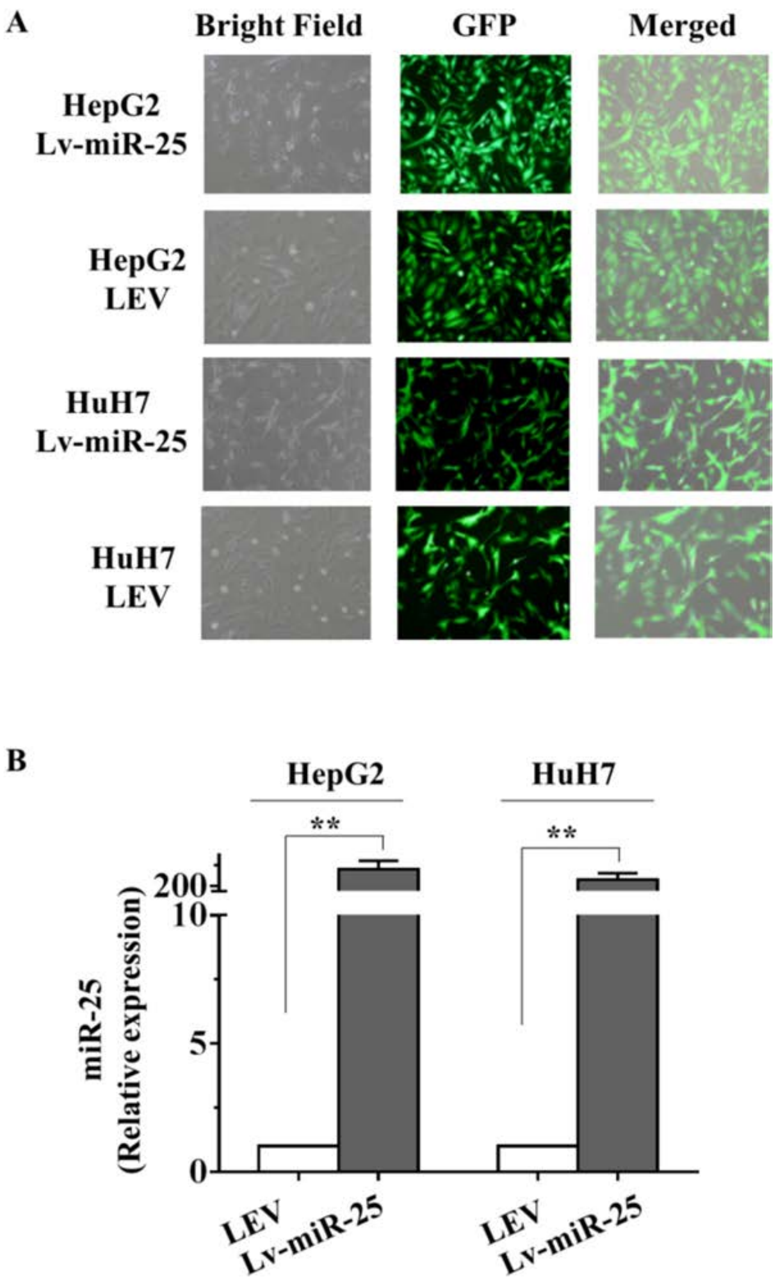

Supplementary Figure S1: miR-25 overexpression lentiviral vector (Lv-miR-25) and controlled lentiviral empty vector were stably transfected into human HCC HepG2 and HuH7 cells. A. Fluorescence microscopy identified that transfection efficiency of Lv-miR-25 in HepG2 and HuH7 was more than 90%. **B.** More than 200-fold increase in the expression of miR-25 was observed in Lv-miR-25-HepG2 cells and Lv-miR-25-HuH7cells compared to the LEV group by qRT-PCR. Data are presented as mean ± SD from three independent experiments. $^{**}P < 0.01$ compared with the LEV control group.

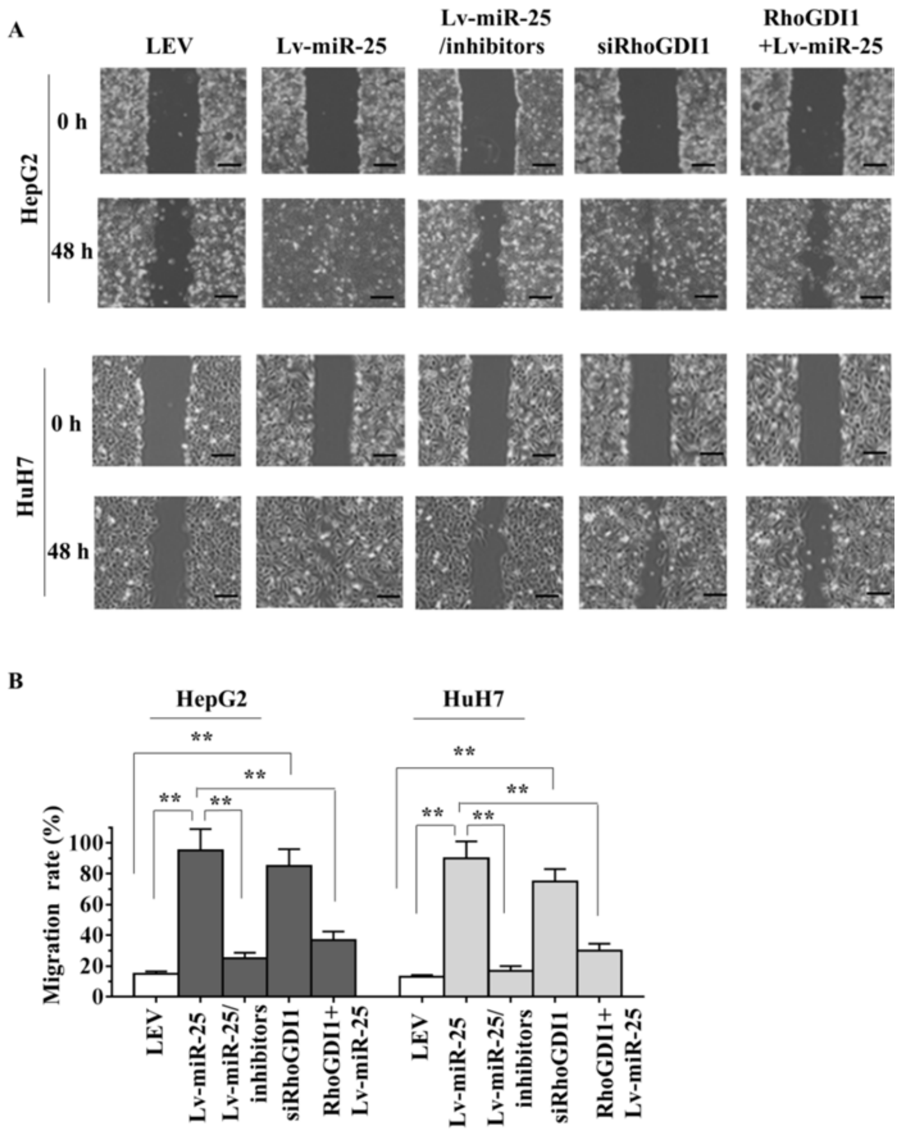

Supplementary Figure S2: Effects of miR-25 overexpression and RhoGDI1 inhibition on the migration of HepG2 or HuH7 cells. A. Scratch wound healing assay was performed after transfection with NC, Lv-miR-25 or siRNA against RhoGDI1 as indicated. **B.** Mobility rate histograms of each group. Data are presented as mean ± SD from three independent experiments. **$p < 0.01$ compared with the control group. Scale bar, 100 µm.

Supplementary Table S1: Oligonucleotides used in this study

Sequence Name	Sequence (5′ to 3′)
**miRNA mimics and inhibitors**	
Hsa-miR-25 mimics (sense)	CAUUGCACUUGUCUCGGUCUGA
RNA duplex control (sense)	UUCUCCGAACGUGUCACGUTT
miR-25 inhibitor	CUCCCUUCUUUCCUCCCGUCUU
miRNA inhibitor NC	CAGUACUUUUGUGUAGUACAA
**Gene knockdown**	
siRNA-RhoGDI1 (sense)	GGAAAGGCGUCAAGAUUGATT
siRNA-β-catenin (sense)	AGCUGAUAUUGAUGGACAGTT
**Gene cloning Primer**	
miR-25 forward	CCAAGCTAGTGCAATGGGCTCACAGGAT
miR-25 reverse	GCTCTAGCTGTACCTGAGCTTGGACTCT
RhoGDI1 forward	CCAAGCTTATGGCTGAGCAGGAGCCCA
RhoGDI1 reverse	GCTCTAGATCAGTCCTTCCAGTCCTTCTT
**3′UTR of RhoGDI1**	
Wide type forward	CTAGTTATGTAATCGTAAGTATTAAAGTTGTAGCTATATTGTGCAATACTTGA
Wide type reverse	AGCTTCTAGTATTGCACAATATACGTACGAAATTAATACTTACGATTACATTA
Mutant forward	CTAGTTATGTAATCGTAAGTATTAAAGTTGTAGCTATATTAACCGAGACTTGA
Mutant reverse	AGCTTCTAGTCTCGGATAATATACGTACGAAATTAATACTTACGATTACATTA
